# Motif Participation by Genes in *E. coli* Transcriptional Networks

**DOI:** 10.3389/fphys.2012.00357

**Published:** 2012-09-24

**Authors:** Michael Mayo, Ahmed F. Abdelzaher, Edward J. Perkins, Preetam Ghosh

**Affiliations:** ^1^Environmental Laboratory, US Army Engineer Research and Development CenterVicksburg, MS, USA; ^2^Department of Computer Science, Virginia Commonwealth UniversityRichmond, VA, USA

**Keywords:** gene regulatory networks, feed-forward loop motif, complex networks, preferential attachment network models, motif centrality

## Abstract

Motifs are patterns of recurring connections among the genes of genetic networks that occur more frequently than would be expected from randomized networks with the same degree sequence. Although the abundance of certain three-node motifs, such as the feed-forward loop, is positively correlated with a networks’ ability to tolerate moderate disruptions to gene expression, little is known regarding the connectivity of individual genes participating in multiple motifs. Using the transcriptional network of the bacterium *Escherichia coli*, we investigate this feature by reconstructing the distribution of genes participating in feed-forward loop motifs from its largest connected network component. We contrast these motif participation distributions with those obtained from model networks built using the preferential attachment mechanism employed by many biological and man-made networks. We report that, although some of these model networks support a motif participation distribution that appears qualitatively similar to that obtained from the bacterium *E. coli*, the probability for a node to support a feed-forward loop motif may instead be strongly influenced by only a few master transcriptional regulators within the network. From these analyses we conclude that such master regulators may be a crucial ingredient to describe coupling among feed-forward loop motifs in transcriptional regulatory networks.

## Introduction

Many natural and engineered systems can be expressed as networks of nodes connected by links, such as interacting genes or communicating sensor motes. For biological systems, autonomous processes drive formation and maintenance of these networks, such as evolutionary pressures on genetic networks (Crombach and Hogeweg, 2008). Genetic networks are particularly interesting, because they are known to tolerate noise in gene expression (Prill et al., 2005), an ability termed robustness (e.g., see, Kitano, 2004 and references therein). Moreover, it was discovered that genetic networks host repeating patterns of smaller subnetworks, termed motifs (Shen-Orr et al., 2002), that occur far more frequently than would be expected in randomized networks with the same degree sequence. These patterns are thought to be the basic building blocks of complex networks (Milo et al., 2002). While much attention has been directed toward the study of their individual functions, both experimentally (e.g., autoregulatory motifs Wu and Rao, 2010) and theoretically (Magnan and Alon, 2003), much less is known relating their coupling and positions within the network to its robustness.

**Figure 1:**
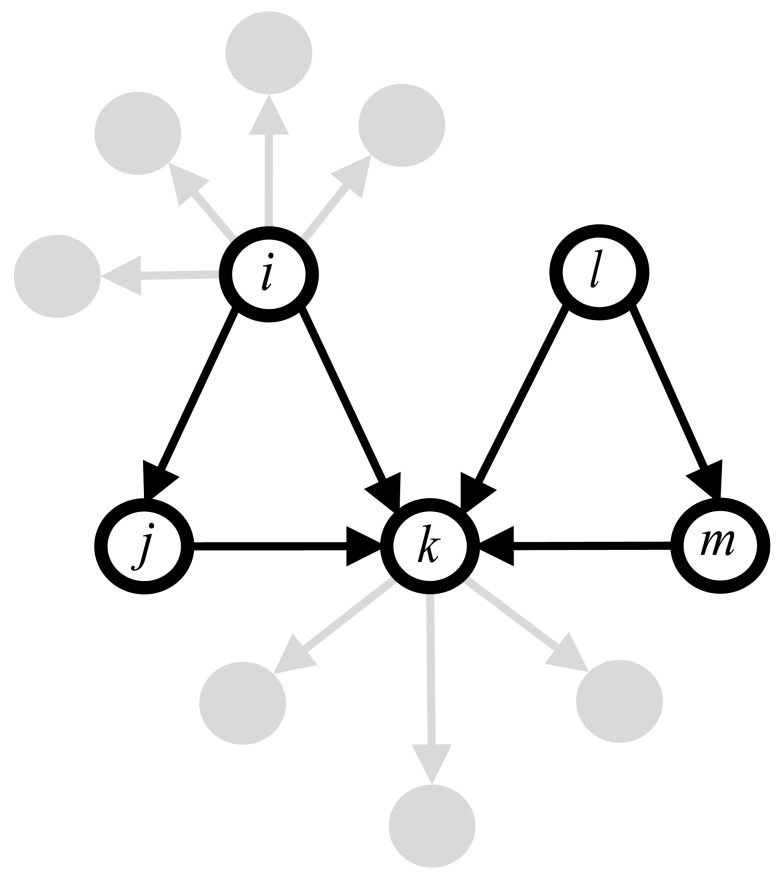
**Genes of feed-forward loops (open circles) may be connected by the participation of common genes**.

Feed-forward loops are one of the most common motifs in genetic networks and are well studied in a variety of biological contexts. In a genetic network, if one gene is linked to another, then it may either enhance or repress the expression level of the target gene, respectively termed up- and down-regulation. A feed-forward loop consists of three genes or nodes, the first of which regulates a second, and both of these co-regulate a third (Figure 1). Recently, Alon and collaborators (Magnan and Alon, 2003) discovered that individual feed-forward loops possess interesting dynamical properties, such as signal delay and pulse generation. Although it is not generally clear how coupling among these motifs affects the overall network function, several groups are beginning to move in this direction. For example, exhaustive experiments with the bacterium *Escherichia coli* (herein *E. coli*), in which 598 gene promoters were altered to “rewire” its genetic network, showed that most of these new connections are tolerated by the bacteria (Isalan et al., 2008). Mathematical modeling of gene transcription and translation has also been used to investigate the relationship between coupling and function among differing motif configurations (Kim et al., 2007; Kwon and Cho, 2008; Wu and Rao, 2010). However, a requisite for using these results to understand complex features at the network level, such as robustness, is a more basic understanding of how motifs are coupled together and distributed throughout such transcriptional networks.

Here we begin to address this problem by measuring the participation of individual genes in each feed-forward loop of the genetic network of the bacterium *E. coli*. We use computational methods to count the number of unique motifs in which a single gene participates, for all genes in the network. To aid in the interpretation of these motif participation distributions, we contrast them with those arising from model networks built using a preferential attachment scheme, employing both linear and non-linear attachment kernels (Krapivsky et al., 2000). The shape of these motif participation distributions contains valuable information that allows us to quantify the extent to which feed-forward loops couple to the whole-network.

## Materials and Methods

### Participation of genes in motifs distributed throughout a network

Here we consider the transcriptional regulatory network of the bacterium *E. coli* as a prototypical genetic network, by which we mean that genes interact with one another when transcription products affect the transactivation of other “target” genes by interacting with their promoter regions. Not only are all connections among genes in *E. coli*’s genetic network well validated by experiments (e.g., see. Shen-Orr et al., 2002), but these data are also easily sampled using the software tool *GeneNetWeaver* (Schaffter et al., 2011), first introduced to aid the development of more accurate gene regulatory network inference algorithms. *E. coli*’s genetic network supports 23 disjoint subnetworks that together form a network of 1565 genes and 3758 links, and it is not completely connected. Based on this observation we restrict our analyses to its largest connected component (LCC), which is sparse, supporting 1477 genes and 3671 directed links.

For each gene in the LCC of this genetic network, we count how many feed-forward loop motifs a gene participates in as one of its three elements, illustrated as nodes *i*, *j*, or *k* in Figure 1. The software tool *mFINDER* (Milo et al., 2002) was used here to identify feed-forward patterns in the network, independent of whether one gene up- or down-regulates another. So we did not distinguish between, for example, coherent and incoherent feed-forward loops in the counting procedure. Motifs were compared to one another to ensure that they were only counted once for each gene. These steps were repeated for the model networks built from procedures described below.

### Degree distributions for growing networks

Because *E. coli*’s LCC is a directed network, it supports two distinct distributions that together describe the total-degree distribution. For a network of *n* nodes, these are (i) the fraction of the network hosting *K*-many outgoing links, *p*(*K*, *R*, *n*), termed the out-degree distribution, and (ii) the fraction of the network hosting *R*-many incoming links, *q*(*K*, *R*, *n*), termed the in-degree distribution.

The growth of several man-made or technological networks, such as citation, internet, actor, and scientific co-authorship networks has been measured before (Jeong et al., 2003), and their growth was modeled by a scheme that adds links to new nodes in a way that depends on the degree of a candidate node of the existing network – a mechanism for network evolution termed preferential attachment (Barabási and Albert, 1999). Although these man-made networks have been observed to “grow” according to preferential attachment, gene networks in *E. coli* and other organisms may instead evolve in response to environmental stressors realized as horizontal gene transfers (Pál et al., 2005) or gene duplication events (Lagomarsino et al., 2007). While these and other mechanisms may indeed drive transcriptional network growth, it remains unclear what role they play in the creation and persistence of genetic motifs. Because preferential attachment offers a simplified view of network growth and has been relatively well studied, we employ it here to develop formulas for the creation of directed networks, wherein the network evolution is determined by an attachment kernel taking one of several forms explained below, either linear, power-law, or sigmoid types.

Consider a network of *n* nodes, wherein each of its nodes labeled by the subscript *i *= 1, 2,…, *n* hosts *K_i_* outgoing links and *R_i_* incoming links. A randomized network is grown by adding nodes one at a time, increasing its size by exactly one node during each round of attachment (also termed a simulation step). These “new” nodes are attached to the existing network by an average of *m* directed links to “candidate” nodes of the network, chosen with equal probability among all existing network nodes. The probability for an edge to link a candidate node *i* with the new one directed from the candidate to the new one is generally given by *A*(*K_i_, R_i_*), wherein *K_i_* and *R_i_* label the out- and in-degrees of the candidate node, respectively. The probability for a link to be drawn from the new node to a candidate node *i* is similarly given by *B*(*K_i_, R_i_*). These probabilities are normalized against all nodes of the existing network, and are termed attachment kernels (Krapivsky et al., 2000).

The number of nodes in the existing network with degree *K* can be written as *np*(*K, n*)*mA*(*K*), wherein *p* and *A* are assumed to be independent of the nodes’ in-degree *R*. Using this expression, a master equation may be written that describes the evolution of this out-degree distribution:

$$\begin{array}{l} \left(n+1\right)p\left(K,n+1\right)-np\left(K,n\right)=np\left(K-1,n\right)mA\left(K-1\right)\\ \;-np\left(K,n\right)mA\left(K\right),\text{ (1)} \end{array}$$

Equation 1 holds for all cases except *K *= *m*, which describes the links extending from the new node to the existing network. For this case we have

(2) $$\left(n+1\right)p\left(m,n+1\right)-np\left(m,n\right)=1-np\left(m,n\right)mA\left(m\right).$$

Equations 1 and 2 are difficult to solve exactly. In light of this difficulty we instead simulated the growth algorithm directly using computational means using attachment kernels listed in Table 1, and described by the algorithm given below. Nevertheless, by using suitable approximations for Eqs 1 and 2 we can infer a general form for the degree distribution; however, the exact relationship reflecting the frequency of degrees observed for network nodes depends strongly on the specific form of the attachment kernel, as demonstrated here.

**Table 1:** **Normalized attachment kernels used to create the model networks**.

Functional type	Attachment kernel (e.g., *A *= *a*/*z*)			
	*a*	*z *= $\sum_{i}$*a_i_*	*b*	*z *= $\sum_{i}$*b_i_*
Linear	*K*	$\sum_{K}$*Kp*(*K*)	*R*	$\sum_{R}$*Rq*(*R*)
Power-law (γ = 0.8)	*K*^γ^	$\sum_{K}$*K^γ^p*(*K*)	*R*^γ^	$\sum_{R}$*R^γ^q*(*R*)
Sigmoid	*K* / (*K *+ *R*)	$\sum_{K}\sum_{R}\frac{Kp\left(K\right)q\left(R\right)}{K+R}$	*R* / (*K *+ *R*)	$\sum_{K}\sum_{R}\frac{Rp\left(K\right)q\left(R\right)}{K+R}$

By taking an approximation valid for very large networks, *n*→∞, we can solve for the degree distribution near this limiting value. Here we label *p*(*K*, ∞) = *p*(*K*), so that (*n *+ 1)*p*(*K*, *n *+ 1) − *np*(*K*, *n*)∼*p*(*K*). Then, Eqs 1 and 2 become (Newman, 2010)

$$\begin{array}{ll} p\left(K\right)=np\left(K-1\right)mA\left(K-1\right)-np\left(K\right)mA\left(K\right),\;\text{and} & \text{(3)}\\ p\left(m\right)=1-np\left(m\right)mA\left(m\right). & \text{(4)} \end{array}$$

As shown in the appendix, Eqs 3 and 4 can be solved to give

(5) $$p\left(K\right)=\frac{1}{nmA\left(K\right)}e^{-\overset{K}{\underset{i=m}{\sum }}1∕nmA\left(i\right)},$$

wherein the attachment kernel is “small,” i.e., for *A*(*K*) > 1/*nm*. Equation 5 can be further reduced when the actual dependence of *A* (or *B*) on the out- or in- degrees is known (for an example, refer to the Appendix).

### Algorithm to generate model networks

Synthetic networks are grown step-wise according to the following protocol. First, a candidate node, denoted by subscript *i* here, is chosen randomly with equi-probability from the existing network of size *n*. Next, a link directed from the candidate node to the new one is drawn if a number selected at random from an equi-probable distribution on the interval *d *∈ (0, 1) generally satisfies *d *≤ *A*(*K_i_*, *R_i_*). This process is then repeated for a link to be drawn from the new node to the candidate, wherein a newly drawn random number from this same distribution instead generally satisfies *d *≤ *B*(*K_i_*, *R_i_*) These steps were repeated *m_i_ *− 1 times, wherein *m_i_* is another number drawn at random, and the final sequence of such numbers after *S* growth steps {*m_l_*:*l *= 1, 2, …, *S*} satisfies the following exponential distribution:

(6) $$\rho \left(m_{i}\right)=\left(f^{1∕\left(1-m_{0}\right)}-1\right)f^{-m_{i}∕\left(1-m_{0}\right)}.$$

Parameters here are chosen so that ρ(*m*_i_ = *m*_0_)/ρ(*m_i_ *= 1) = *f*, with the values *f *= 1/4 and *m*_0_ varied for creation of the model networks between 2, 3, and 4, which skews the distribution toward larger values of average *m_i_*. The average number of links chosen per growth step, *m*, is given in terms of these parameters as

$$m=\overset{\infty }{\underset{m_{i}=1}{\sum }}m_{i}\rho \left(m_{i}\right)=\frac{1}{1-f^{1∕\left(m_{0}-1\right)}}.$$

So, in view of this expression the average number of links supported by model networks built using *m_i_ *= 2, 3, and 4 is approximately *m *= 1.33, 2, and 2.7, respectively.

The form of this distribution of link enumerations, Eq. 6, was chosen partly because the majority of *E. coli*’s genes support only 1 or 2 links, rather than many more. Computer experiments using other link distributions, such as *m_i_ *= constant, generated motif participation distributions in greater variance with the *E. coli* distributions than generated using Eq. 6 (data not shown here). We note that model networks were built over a “seed” network of eight nodes fully connected supporting 42 links. This ensures that early in the growth process, when the network is “small,” it is much less likely for values of *m_i_* to force the creation of duplicate links. That is, more than one link of the same direction connecting two nodes is not permitted.

### Choice of the attachment kernels

As evidenced by Eq. 5, the dependence of the attachment kernel on the degree determines the ultimate shape of the in- or out-degree distribution. In a celebrated publication (Barabási and Albert, 1999), Barabási and Albert demonstrated how a variation of the “Matthew effect” – the idea that already-famous individuals are awarded credit disproportionately (Merton, 1968) – can be employed to generate model networks presenting power-law tails in their degree distributions. In the attachment kernel formalism of evolving networks (Krapivsky et al., 2000, 2003; Krapivsky and Redner, 2001), the Barabási–Albert model is equivalent to an attachment kernel that is linear in the node degree. Because it is well known that *E. coli* supports degree distributions presenting similar power-law type distributions (Shen-Orr et al., 2002) we employ it here to generate model networks.

Until recently, network evolution was investigated primarily by studying the growth of model networks possessing qualities similar to biological and technological (i.e., man-made) networks. While direct study of growth regarding some networks, such as the internet (e.g., Pastor-Satorras et al., 2001), has been conducted on a limited scale by measuring properties at discrete time points, only recently has a direct measurement of the attachment kernel been made as the network continues to grow. Building on previous works (Newman, 2001; Barabási et al., 2002), Jeong et al. (2003) inferred the form of these kernels by employing a best fit statistical method to the network of co-authorship among scientists working in neuroscience (between the years 1991 and 1998); the citation network between published papers in the journal Physical Review Letters beginning from the year 1998; a collaboration network among actors appearing in the same movie and debuting between 1920 and 1940 and continuing through to 1993; finally, to the internet beginning with the year 1997. While it is clear that these networks do not all grow identically, e.g., actors die or retire while citation records remain immutable, all of these networks were found to grow according to preferential attachment. In particular, attachment kernels for these networks are well described with a power-law of exponent ∼1 (internet and citation networks) or ∼0.8 (actor and collaboration networks). While we consider network models using the former kernel by using the Barabási–Albert model of (directed) linear preferential attachment as described above, we additionally use here model networks built using power-law kernels with exponent 0.8 to contrast the motif participation networks derived from *E. coli*. Data supporting these results has been recently reported to arise in networks of Wikipedia pages (Capocci et al., 2006).

It is possible for evolved networks to have been created dynamically according to preferential attachment under evolutionary conditions – a conclusion based on data obtained from protein-interaction networks of yeast (*Saccharomyces cerevisiae*) evolving under gene duplication events (Eisenberg and Levanon, 2003; Wagner, 2003; Berg et al., 2004). So, preferential attachment leading to power-law type networks may provide a reasonable model of network growth over such long time scales. We explore the implications of a strongly non-linear attachment kernel on network growth, which we consider to be proportional to the ratio of either the out-degree or the in-degree to a nodes’ total-degree: *K*/(*K *+ *R*) or *R*/(*K *+ *R*), respectively. Under this hypothesis, nodes have a tendency to support incoming or outgoing edges relative to its total-degree, which is a manifestly local feature of these nodes. As an example, we note that sigmoid type growth kinetics are ubiquitous throughout biochemical networks, commonly used to model the yield of enzyme-mediated reactions that create or degrade biomolecules.

### Probability for a node to participate in a feed-forward loop

As shown in the appendix, the number of feed-forward loop motifs that a node with *K* outgoing links and *R* incoming links supports is proportional to the probability that a node participates in a feed-forward loop motif, *p*_motif_(*K*, *R*), which is given by the following formula (Eq. A13 of the Appendix):

$$\begin{array}{ll} p_{\text{motif}}\left(K,R\right)=p\left(K\right)q\left(R\right)\frac{n^{2}}{L^{3}}\left[K^{2}\left\langle R^{2}\right\rangle \left\langle KR\right\rangle +R^{2}\left\langle K^{2}\right\rangle \left\langle KR\right\rangle \right.\\ \left.\;+KR\left\langle K^{2}\right\rangle \left\langle R^{2}\right\rangle \right]. & \text{(7)} \end{array}$$

Note that Eq. 7 is a function of *K* and *R*, and cannot be directly compared to the result of the motif counting procedure directly, which relates how many nodes host a particular number of motifs.

### Maximum likelihood estimation of cumulative distribution functions

Many features of interest in biology when subjected to repeated measurement show a cumulative probability distribution that follows power-law type mathematical relationship (Clauset et al., 2009). For reasons discussed above, the in-, out-, or total-degree distributions of a network may support a power-law type tail depending on the form of the attachment kernel used to build it (e.g., Eq. 5). However if there are no *a priori* theoretical considerations to predict whether experimental data should best fit to a particular distribution, then curve-fitting methodologies are commonly used to justify empirical relationships among features in these data. It is known, for example, that using a least squares based optimization algorithm does not accurately determine whether the data are power-law distributed (Hoogenboom et al., 2006; Clauset et al., 2009).

Addressing this problem, Hoogenboom et al. (2006) presented a maximum likelihood estimation based approach that determines whether data are power-law distributed or not. For illustration, let *p*(*K*;γ) be an out-degree distribution function that depends on a parameter γ, such as *p*(*K*;γ)∼*K*^−γ^. A likelihood function is then defined from this distribution so that $L\left(\gamma \right)=\underset{K}{\prod }p\left(K;\gamma \right)$. To find the parameter γ that best fits the experimental data, this likelihood function is maximized with respect to it. To carry out these analyses on the motif participation and degree distributions extracted from the experimental and synthetic networks described above, we employed MATLAB implementations of the maximum likelihood estimation method of Hoogenboom et al. as described by Clauset et al. (2009).

## Results and Discussion

### Cumulative degree distributions

**Figure 2:**
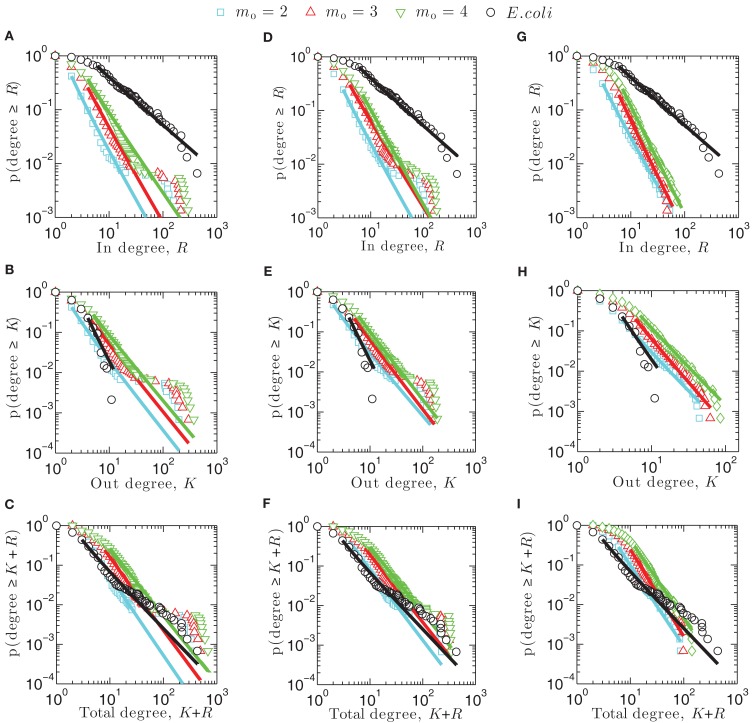
**Cumulative degree distributions for synthetic networks created using linear attachment kernels (A–C), power-law kernels (D–F), and sigmoidal type kernels (G–I)**.

Figure 2 illustrates the cumulative degree distributions of one representative network generated computationally using the attachment kernels listed in Table 1 for varying distributions of the link enumeration as given by Eq. 6, contrasted against the associated distributions arising from the *E. coli* network (black circles). Straight lines are the result of the maximum likelihood estimation of the validity of a power-law fit to these cumulative distributions, *p*(degree ≥ *K*), which measures, for example, the probability that observation of the out-degree for any network node is greater than *K*. The cumulative distribution is related to the degree distribution, *p*(*K*), by the equation

(8) $$p\left(\text{degree}\geq K\right)=\overset{\infty }{\underset{i=K}{\sum }}p\left(i\right).$$

Similar equations exist relating in and total-degree distributions to their associated cumulative distributions.

In, out, and total cumulative degree distributions arising from the linear attachment kernel are displayed here in Figures 2A–C. Notably, scaling exponents for power-law type equations fit to these distributions, such as *p*(degree ≥ *K*) ∼ *K*^α^, do not differ greatly between *m*_0 _= 2, 3, or 4; exponents are collected for *m*_0 _= 2 networks (cyan in Figure 2) into Table 2. A point-wise inspection of the cumulative total-degree distribution over its whole domain *K *+ *R*, however, closely resembles that for *E. coli* (Figure 2C), while the cumulative in- and out-degree distributions do not match qualitatively with *E. coli* very well. This observation is consistent with power-law (Figures 2D–F) and sigmoidal (Figures 2G–I) attachment kernel constructed networks.

**Table 2:** **Scaling exponents α, defined for cumulative distribution functions *p*(feature ≥*x*) ∼ *x*^−^^α^, identified using the maximum likelihood fitting procedure explained in the section “Materials and Methods” for the degree and motif participation distributions illustrated in Figures 2 and 3**.

Distributions		Features			
		In-degree	Out-degree	Total-degree	Motif
Model networks	Linear	2.7671	2.6973	2.9765	1.8289
	Power-law	2.7452	2.5378	2.7373	1.8484
	Sigmoid	2.7462	2.8632	2.9953	1.8856
Experimental network	*E. coli*	1.871	3.4922	2.4078	2.0079

*Data are collected here for networks with *m*_0 _= 2*.

**Figure 3:**
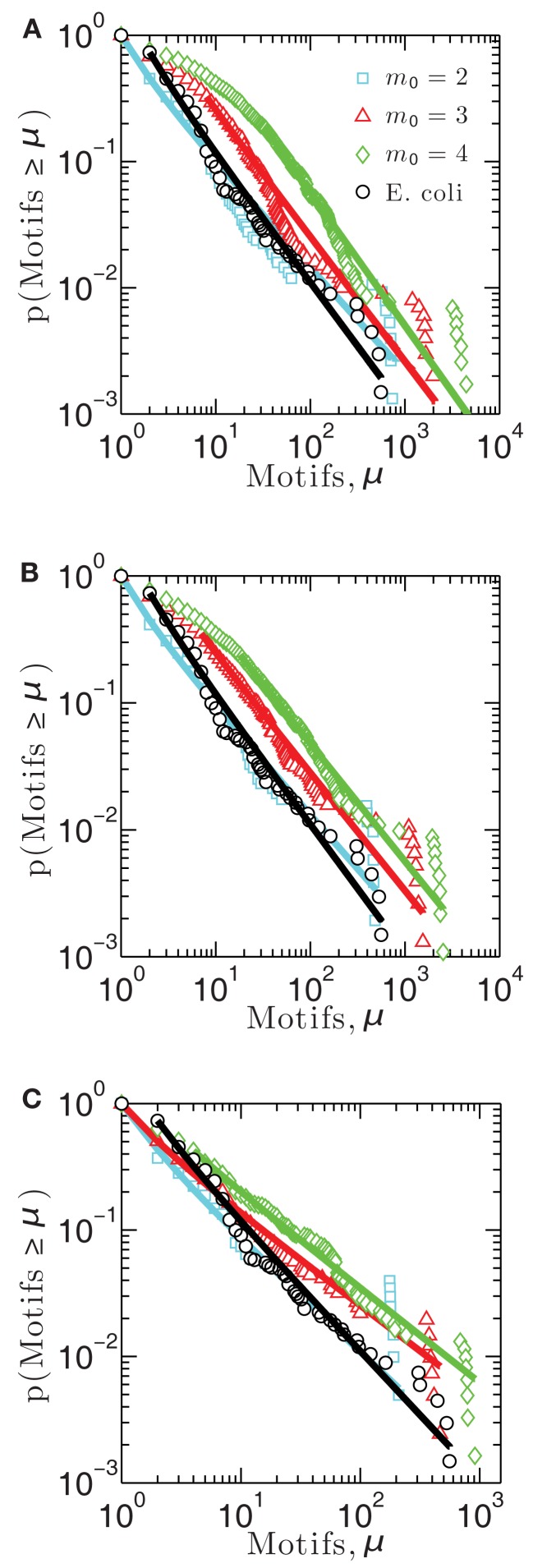
**Cumulative distribution functions measuring the probability that a measurement made on a network node gives a number of motifs greater than μ, for each of three model networks built using (A) linear attachment kernels, (B) power-law kernels, and (C) sigmoidal kernels**.

As expected, when more links are added (e.g., *m*_0 _= 4) the distributions illustrated in Figure 2 are shifted more toward the right, demonstrating that nodes of such networks support larger degrees merely because the density of links has increased. The form of these distributions remains similar, however, appearing to be mostly independent of the choice of *m*_0_. For example, in networks built using the linear (Figures 2A–C) and power-law (Figures 2D–F) attachment kernels, a plateau arises in the cumulative distribution that persists across a decade or so in each degree type. This plateau describes a region in the degree (i.e., *x*-axes of Figure 2) for which there is constant probability that a measurement of a node’s degree gives a greater value than the considered one. Given the definition of the cumulative distribution, Eq. 8, the existence of the plateaus mean the degree distributions for the model power-law networks are bimodal, with a longer plateau indicative of a longer span in the degree between maxima of the degree distribution.

The cumulative total-degree distribution for *E. coli*, illustrated by black circles Figures 2C,F,I, begins to moderately vary from the power-law fit obtained from the maximum likelihood estimation method (black line) at approximately *K* + *R* = 20, lasting until approximately *K* + *R* = 200. This variance is not strictly indicative of a plateau, but does hint that power-law-type factors may be ingredients in the evolutionary pressures leading to the shape of the final transcriptional network distribution. Interestingly, preferential attachment mechanisms have indeed been suggested for this purpose yielding scale-free protein-interaction networks (see, e.g., Barabási and Oltvai, 2004). It was also shown that highly connected genes evolve more slowly (and are therefore older) than their loosely connected peers and that co-expressed genes evolve at similar rates (Jordan et al., 2004). (There are, however, exceptions to this conclusion in the case of protein-interaction networks, e.g., Kunin et al., 2004.) These data suggest preferential attachment contributes to transcriptional network evolution, lending weight to our conclusion that a moderate departure from scale-free topology observed in the *E. coli* (Figures 2C,F,I) cumulative total-degree distribution data is consistent with a power-law-type preferential attachment growth mechanism. However, the reason even minor bimodality should present in the *E. coli* transcriptional network topology remains unknown.

### Participation of *E. coli* genes in feed-forward loop motifs

Figure 3 illustrates the cumulative motif participation distributions for networks constructed using each of the three attachment kernels: linear (Figure 3A), power-law (Figure 3B), and sigmoid (Figure 3C). As with the distributions of Figure 2, scaling exponents for these motif participation distributions are also collected into Table 2.

As the number of motifs associated with a node, μ, increases, the probability that a node will host a greater number of such motifs decreases for all networks (Figures 3A–C) – a result consistent with the *E. coli* data (depicted with black circles). As expected, when more links are added on average per growth step (i.e., increasing *m*_0_), or more generally as the network density increases, feed-forward loop motifs are more likely to be created by the attachment procedure. This is the reason these cumulative motif participation distributions mostly shift toward the right in Figures 3A–C with increasing *m*_0_. While differences between the cumulative distribution scaling exponents for these representative networks built using *m*_0 _= 2 and *E. coli*’s motif participation distribution are the largest of any *m*_0_ values considered here, these *m*_0 _= 2 networks nevertheless more closely resemble the overall *E. coli* motif distribution. Of these, the *m*_0 _= 2 network of Figure 3A provides the closest match to the *E. coli* data for the kernels considered here.

### Feed-forward loop motif probability

**Figure 4:**
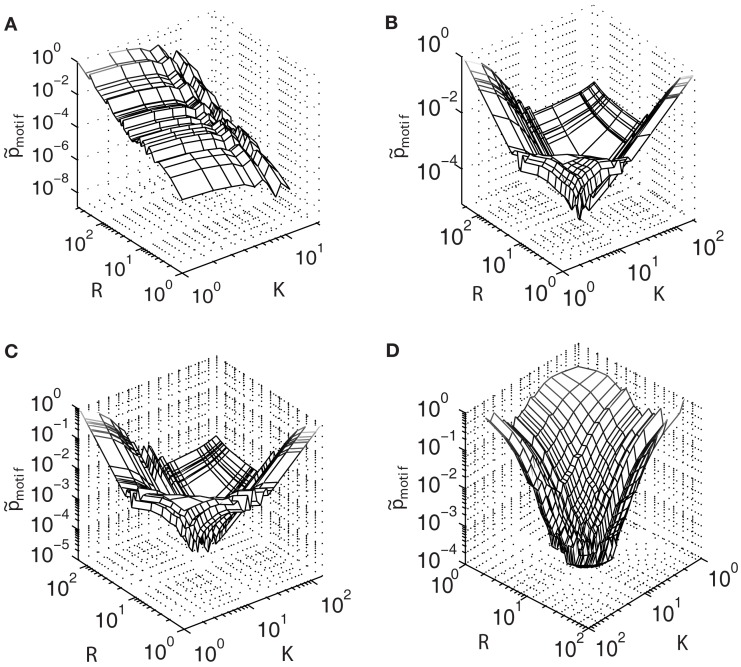
**Motif probability distributions (Eq. 6) for the (A) largest connected component of the *E. coli* transcriptional network, contrasted with distributions obtained from model networks built using (B) linear attachment kernels, (C) power-law kernels, and (D) sigmoidal type kernels**.

Another way to understand the overlap of feed-forward loop motifs among network nodes is to determine how likely a node is to participate in such a feed-forward loop motif. This quantity can be computed directly from the degree distributions of the networks we have considered here, which is given by Eq. 7 above. So, for any given pair of in- and out-degrees, Eq. 7 returns the probability that a node will not only possess those values, but will also participate in a feed-forward loop motif, playing the part of any of its three nodes *i*, *j*, or *k* (as depicted in Figure 1). Figure 4 reports these probability distributions using degree distributions obtained computationally from the experimental and synthetic preferential attachment networks, as a function of *K* and *R*, but scaled so that their global maximum is unity. Distributions so scaled are denoted by ${\tilde{p}}_{\text{motif}}\left(K,R\right).$

Measured in this way, it is clear that the motif structure of *E. coli* (Figure 4A) is qualitatively very different from that of the model networks (Figures 4B–D), in direct contrast to the similarity observed between the motif participation distributions illustrated in Figure 3. For *E. coli*, the probability to find a motif in the network is greatest for larger values of *R* (but smaller values of *K*). This feature may arise because nodes possessing larger degrees will be more strongly coupled with the rest of the network. In this view, a higher density network is favorable because it is more abundant in motifs. However, this explanation cannot be the whole story, because the maximum probability occurs also when *K* is minimal.

This asymmetry between the influence of the out- and in-degrees on the motif probability suggests that biological mechanisms driving the evolution of the degree distributions are themselves asymmetric, favoring one over the other. One way for this to occur is if genes are more frequently regulated than are actively regulating other genes. Such “master regulators” are known to exist in the *E. coli* network (Babu and Teichmann, 2002). In view of this evidence, in-degrees should be more frequent within the network; however, if most genes are not actively regulating other ones, then there are many more combinations of genes with high in-degree but low out-degree. A consequence of this regulatory strategy is that genes with higher in-degree but lower out-degree are more likely to participate in a feed-forward loop motif – a result that may be responsible for the global maximum at small *K* but large *R* in Figure 4A.

This “regulatory asymmetry” is not an ingredient in the model networks, which treat the building of the in- and out-degrees equally because the form of the attachment kernels is the same for both in- and out-degree distributions. This fact manifests as the symmetry observed in the motif probability distributions shown in Figures 4B–D. Especially interesting is the existence of a global minimum at values intermediate to the minimum and maximum out- and in-degrees of model networks built using linear and power-law type attachment kernels (Figures 4B,C).

The global minimum observed at intermediate values of *K* and *R* in Figures 4B,C results from the bimodality of the power-law type cumulative in- and out-degree distributions (Figures 2A–F), because these plateaus describe a local minima in the degree distributions. However, networks built using sigmoid type attachment kernels (Figure 4D) exhibit no such distinctive plateaus (Figures 2G–I). Indeed the global minimum of the motif probability distribution for the sigmoid based preferential attachment network (Figure 4D) occurs at maximum *K* and *R*, which might result directly from the attachment kernel: nodes with many in-degrees (larger *R*), which are already rare (Figure 2G), may be less likely to also support many out-degrees (larger *K*), and therefore be unlikely to support the links necessary to form a complete feed-forward loop motif. In this case, the more loosely connected nodes stand the best chance of participating in a feed-forward loop motif merely because they are more likely to acquire both in- and out-degrees during the preferential attachment process.

### Motif participation centrality and modularity

The idea that a gene of a transcriptional network can be ordered according to its motif participation suggests that we may also use these distributions as a way to define a measure of their network centrality. Following ideas introduced by Koschützki et al. (2007), Koschützki and Schreiber (2008), we define here motif centrality as the number of motifs associated with a gene. The motif participation distributions are then used to obtain a sequential ranking for each gene in the LCC of *E. coli*’s transcriptional network.

Ranking genes according to their feed-forward loop motif participation reveals that five *E. coli* genes support greater than 300 feed-forward loop motifs, while the majority of genes are mostly insulated from the network by participating in just a few feed-forward loop motifs. The top five genes are listed in Table 3, and we find they are all transcription factors, with the single exception of one receptor. This result supports the idea that only a few master regulators are in feed-forward loop motifs within the *E. coli* transcriptional network. We note that the 6th gene in this motif-participation hierarchy, arcA, participates in approximately 50% of the number of motifs of the 5th gene, fis: 163, and serves as a natural cutoff between the top-ranked genes and the rest of the network.

**Table 3:** **Top five genes in the motif participation distribution**.

Gene	Description	No. motifs
ihfA	Transcription factor	559
ihfB	Transcription factor	529
crp	cAMP receptor protein	378
fnr	Global transcription factor for anaerobic growth	316
fis	Transcription factor	307

While we have not examined the clustering relationships among the motifs themselves, it is already known that feed-forward loops do not exist in isolation in the *E. coli* transcriptional network, but rather exist within modules of higher motif density and connectivity through overlapping genes composing these motifs (Dobrin et al., 2004). Because Figure 3 demonstrates that only a few genes disproportionately support many motifs, while many genes support only a few motifs, we hypothesize that these few genes are more likely to reside in motif modules of higher density. Therefore, such genes may prove to be sensitive genes for metrics relying on an optimal connectivity among them, possibly such as network robustness.

## Conclusion

Motifs are thought to be the elementary building blocks of complex biological networks, because they are attributed special functions not present in the nodes themselves. For example, feed-forward loop motifs can delay signal transmissions or assist with pulsing behavior when isolated (Magnan and Alon, 2003). Frustrating attempts to understand the role of coupled motifs in *E. coli* and other transcriptional networks is that some feed-forward loops may be more or less important than others; specifically, it has been shown before that not all feed-forward loop motifs are equally unexpected when compared against certain randomized networks (Camas and Poyatos, 2008). Because feed-forward loop motifs within transcriptional networks do not reside in isolation (Dobrin et al., 2004), they may be organized into compartments of high density.

Although we did not directly measure such an organization of motifs in the networks we considered, genes participating in many motifs (Table 3) are more likely to reside in such compartments. Additionally, the power-law motif participation distribution we report above (Figure 3) demonstrates that only a few genes are integrated throughout the motif network structure; perturbing the expression patterns of these genes should therefore influence network metrics that rely on the large-scale connectivity among motifs. This should be contrasted with the majority of genes, which are insulated from most other motifs by low participation. Because it is known that the abundance of feed-forward loop motifs is positively correlated with network robustness as measured by noise reduction in gene expression (Prill et al., 2005), genes contributing to a larger number of motifs may provide natural targets of future studies investigating this connection.

Finally, measuring the motif probability (Eq. 7) of *E. coli* suggests that a small number of master transcriptional regulators are important elements in the distribution of feed-forward loops. Such regulators are not normally considered when building randomized networks; we have shown that such networks support feed-forward loop distributions that poorly reflect the biological foundations of the *E. coli* transcriptional network. Because the connectivity between these master regulators and the rest of the network may strongly contribute to the motif probability distribution (Figure 4A), the effect that such regulators contribute to network functionality should be added as an ingredient in future models that hope to realistically describe the coupling among and distribution of feed-forward loop motifs.

## Conflict of Interest Statement

The authors declare that the research was conducted in the absence of any commercial or financial relationships that could be construed as a potential conflict of interest.

## Appendix

### The in- and out-degree distributions

Here we find a solution for the out-degree distribution as given by Eqs 3 and 4. The derivation to obtain a formula for the in-degree distribution is exactly the same, assuming that the in-degree attachment kernel is independent of a nodes’ out-degree. Therefore, we restrict ourselves to solving only Eqs 3 and 4. Note that this derivation follows one provided in detail by Newman (2010).

Master equations are given for the out-degree distribution approximated for very large networks (Eqs 3 and 4):

$$\begin{array}{rc} p\left(K\right)=np\left(K-1\right)mA\left(K-1\right)-np\left(K\right)mA\left(K\right),\;\text{and} & \text{(A1)}\\ p\left(m\right)=1-np\left(m\right)mA\left(m\right). & \text{(A2)} \end{array}$$

Equation A1 can be written as a recursion between out-degrees *K* and *K−*1:

$$p\left(K\right)=\frac{nmA\left(K-1\right)}{1+nmA\left(K\right)}p\left(K-1\right).$$

Iterating this recursive relationship, and including Eq. A2, gives

(A3) $$p\left(K\right)=\frac{1}{nmA\left(K\right)}\overset{K}{\underset{i=m}{\prod }}{\left[1+\frac{1}{nmA\left(i\right)}\right]}^{-1}.$$

Now, using the fact that *x *= *e*^ln*x*^, Eq. A3 can be rewritten as

(A4) $$p\left(K\right)=\frac{1}{nmA\left(K\right)}e^{-\overset{K}{\underset{i=m}{\sum }}\ln\left[1+1∕nmA\left(i\right)\right]}.$$

Equation A4 may be further reduced. By assuming the quantity 1/*nmA*(*i*) is “small” (i.e., *A*(*K*) > 1/*nm*), we may expand ln[1 + 1/*nmA*(*i*)] about 1/*nmA*(*i*) = 0 in a Taylor series:

$$\ln\left[1+1∕nmA\left(i\right)\right]=1∕nmA\left(i\right)-{\left[1∕nmA\left(i\right)\right]}^{2}∕2+\cdots \;.$$

Keeping the leading order term and putting this back into (Eq. A4) gives:

(A5) $$p\left(K\right)=\frac{1}{nmA\left(K\right)}e^{-\overset{K}{\underset{i=m}{\sum }}1∕nmA\left(i\right)}.$$

Depending on the relationship between *A* and *K*, the summation in Eq. A5 may either be exact or further approximated. Because Jeong et al. (2003) measured γ = 0.8 for many real-world networks, we have used this distribution as a choice of synthetic network in the main text. This special case of *nA*(*K*) = *K*^γ^/*z* for 1/2 < γ < 1 [with normalization condition *z *= ∑*K*^γ^*p*(*K*)] was solved along with other cases in Krapivsky et al. (2000); see also Newman (2010):

(A6) $$p\left(K\right)∽K^{-\gamma }e^{-zK^{1-\gamma }∕m\left(1-\gamma \right)}.$$

### The probability that a feed-forward loop is associated with a node

Here we calculate the probability that a network node participates in a feed-forward loop motif in a directed network with arbitrary in- and out-degree sequences. Suppose we choose a node with out-degree *K_i_* and in-degree *R_i_*. The probability, *p_i→j_*, to find a link directed from node *i* to a node *j* can be estimated following an argument presented in Itzkovitz et al. (2003), which we summarize here.

First, the probability to find *no edge* from node *i* to *j* is given by

(A7) $$p_{i\to j}^{\text{no edge}}=\overset{K_{i}}{\underset{l=1}{\prod }}\left[1-\frac{R_{j}}{L-R_{i}-\overset{l}{\underset{m=1}{\sum }}R_{\sigma_{m}}}\right],$$

wherein the target for each outgoing link of node *i* may be contained in a particular set of nodes {σ*_i_*:*i *= 1,…,*k*} that does not include the target *j*. The total probability to find an edge from *i* to *j*, *p_i→*j*_*, is therefore the complement of a sum over all possible ordered sets {*σ_i_*}:

(A8) $$p_{i\to j}=1-\frac{1}{K_{i}!\left(\begin{array}{c} N-2\\ K_{i} \end{array}\right)}\underset{\left\{\sigma \right\}}{\sum }\overset{K_{i}}{\underset{l=1}{\prod }}\left[1-\frac{R_{j}}{L-R_{i}-\overset{l}{\underset{m=1}{\sum }}R_{\sigma_{m}}}\right].$$

This expression, (A8), can be simplified by assuming that Σ*R*_σ_ + *R_i_* « *L* (Itzkovitz et al., 2003), under which Eq. A7 gives

(A9) $$p_{i\to j}=1-{\left(1-\frac{R_{j}}{L}\right)}^{K_{i}}=1-e^{-K_{i}R_{j}∕L}∽\frac{K_{i}R_{j}}{L}.$$

The final approximation made above assumes that the network is sparse: *K_i_ R_j_* ll *L*, a feature that is generally true of biological networks (Leclerc, 2008; Del Genio et al., 2011). The result (Eq. A9) has a very natural interpretation: a link between nodes *i* and *j* might be established after *K_i_*-many independent attempts to hit a target *j* of size *R_j_*/*L*.

To compute the number of feed-forward loops motifs associated with each, we refer to Figure 1. When counting feed-forward loops we do not distinguish between up- or down-regulated edges. A network node may participate in a feed-forward loop as any of the three nodes *i*, *j*, or *k* illustrated by Figure 1. Given that a node hosts out-degree *K* and in-degree *R*, the probability that a node plays the role of *i* in Figure 1, $p_{\text{motif}}^{i},$ is given by

$$p_{\text{motif}}^{i}=\overset{n}{\underset{j=1}{\sum }}\overset{n}{\underset{k=1}{\sum }}p_{i\to j}p_{i\to k}p_{j\to k}.$$

Using Eq. A9, this calculation can be carried out to find

(A10) $$p_{\text{motif}}^{i}\left(K\right)=\frac{n^{2}}{L^{3}}K^{2}\left\langle R^{2}\right\rangle \left\langle KR\right\rangle ,$$

wherein we have used $\frac{1}{n}\sum_{i=1}^{n}K_{i}=\sum_{i=1}^{L}Kp\left(K,n\right)=\left\langle K\right\rangle$ or its equivalent. Similar calculations for the other two nodes yield

$$\begin{array}{rc} p_{\text{motif}}^{j}\left(K,R\right)=\frac{n^{2}}{L^{3}}KR\left\langle K^{2}\right\rangle \left\langle R^{2}\right\rangle ,\;\text{and} & \text{(A11)}\\ p_{\text{motif}}^{k}\left(R\right)=\frac{n^{2}}{L^{3}}R^{2}\left\langle K^{2}\right\rangle \left\langle KR\right\rangle . & \text{(A12)} \end{array}$$

The probability that a node plays the part of any of the three motif nodes, *p*_motif_(*K*, *R*), is given by the sum of (Eqs A10–A12) multiplied by the probability that the node carries the values *K* and *R*, *p*(*K*) and *q*(*R*), respectively. Carrying this out yields

(A13) $$p_{\text{motif}}\left(K,R\right)=p\left(K\right)q\left(R\right)\frac{n^{2}}{L^{3}}\left[K^{2}\left\langle R^{2}\right\rangle \left\langle KR\right\rangle +R^{2}\left\langle K^{2}\right\rangle \left\langle KR\right\rangle +KR\left\langle K^{2}\right\rangle \left\langle R^{2}\right\rangle \right].$$

Note the number of expected motifs for a node with *K* and *R* out- and in-degrees, μ, is proportional to Eq. A13. This proportionality constant is different from the abundance of motifs in the entire network, which is the sum number of all unique motifs distributed across it. For example, Itzkovitz et al. (2003) approximate this abundance for the feed-forward loop in a sparse network:

(A14) $$\frac{\left\langle K\left(K-1\right)\right\rangle \left\langle RK\right\rangle \left\langle R\left(R-1\right)\right\rangle }{{\left\langle K\right\rangle }^{3}}.$$
